# Palliative care competencies for geriatricians across Europe: a Delphi consensus study

**DOI:** 10.1007/s41999-020-00445-5

**Published:** 2021-02-01

**Authors:** Sophie Pautex, Regina Roller-Wirnsberger, Katrin Singler, Nele Van den Noortgate, the SIG palliative care, SIG education of the European Geriatric Medicine Society (EuGMS)

**Affiliations:** 1 Palliative Medicine Division, Department of Rehabilitation and Geriatrics, Geneva University Hospitals, University of Geneva, Geneva, Switzerland; 2 Department of Internal Medicine Graz, Medical University of Graz, Graz, Austria; 3 Department of Geriatrics, Klinikum Nürnberg, Paracelsus Medical University and Institute for Biomedicine of Ageing, Friedrich-Alexander University Erlangen, Erlangen, Germany; 4 Department of Geriatric Medicine, Ghent University Hospital, Ghent, Belgium; 5 Palliative Medicine Division, Hôpital de Bellerive, 11, Ch de La Savonnière, 1245 Collonge-Bellerive, Switzerland

**Keywords:** Palliative care, Postgraduate training, Geriatric medicine, Consensus, Curriculum, European Union, Older people

## Abstract

**Purpose:**

Integration of palliative care competencies with geriatric medicine is important for quality of care for older people, especially in the last years of their life. Therefore, knowledge and skills about palliative care for older people should be mandatory for geriatricians. The European Geriatric Medicine Society (EuGMS) has launched a postgraduate curriculum for geriatric medicine recently.

**Aim:**

Based on this work, the Special Interest Group (SIG) on Palliative care in collaboration with the SIG in Education and Training aimed to develop a set of specific palliative care competencies to be recommended for training at a postgraduate level.

**Methods:**

Competencies were defined using a modified Delphi technique based upon a Likert like rating scale. A template to kick off the first round and including 46 items was developed based on pre-existing competencies developed in Switzerland and Belgium.

**Results:**

Three Delphi rounds were necessary to achieve full consensus. Experts came from 12 EU countries. In the first round, the wording of 13 competencies and the content of 10 competencies were modified. We deleted or merged ten competencies, mainly because they were not specific enough. At the end of the 2nd round, one competence was deleted and for three questions the wordings were modified. These modifications had the agreement of the participants during the last round.

**Conclusion:**

A list of 35 palliative care competencies for geriatricians is now available for implementation in European countries.

## Key summary points

**Aim:**

to develop a set of specific palliative care competencies to be recommended for training at a postgraduate level of geriatricians across Europe.

**Findings:**

A list of 35 palliative care competencies for geriatricians is now available for implementation in the different European countries.

**Message:**

Future action are needed to support implementation and evaluation of the recommendations based upon key performance indicators within different health care systems.

## Introduction

The increase in persons older than age 65 years of which 75% have multiple non-communicable chronic diseases (NCCDs), result in the need for integrated health care [1]. The time spent living with NCCDs is often challenged by intermittent acute illness and progressive loss of functioning, resulting in reduced quality of life [2]. Causes of death therefore shift and the characteristics of the “dying phase" change accordingly”. The later stages of life extend to a longer phase compared to former times and are characterized by challenging treatment decisions due to complex bio-psycho-social care needs, including symptom management, manifold psychosocial problems and easily overlooked spiritual distress.

Several studies have shown the benefit of adopting palliative care approaches in these aging patients [3–5]. Health care professionals equipped with competences and skills in both geriatric and palliative care medicine are capable of delivering personalized and tailored care up to end of life for frail older people. Geriatricians are very involved in care coordination and management of complex care needs of older patients. They often follow-up their patients and liaise with relatives until death. Given this scenario geriatricians also need to be trained in palliative care competences [6, 7].

The European Union of Medical Specialists- Geriatric Medicine Section (UEMS-GMS in collaboration with the European Geriatric Medicine Society (EuGMS) has launched a postgraduate curriculum for geriatric medicine recently [8]. To further specify the palliative care competences included in this curriculum, the members of the EuGMS special interest groups (SIG) on Palliative care in collaboration jointly with members of the SIG in Education and Training aimed Training aimed to develop a set of detailed palliative care competencies at a postgraduate level in Geriatric Medicine. The current paper describes the process, how palliative care competences for postgraduate geriatric specialist training were developed and presents the final recommendations developed during the process.

## Methods

Using a similar recent procedure for developing recommendations for postgraduate training in Geriatric Medicine, the new recommendations for palliative care competences to be included in post-graduate specialist training for geriatricians were developed using a modified Delphi technique [9–11]. The Delphi technique is a well-recognized consensus method used to determine the extent of agreement on an issue. The process generally includes the formation of a template for further rating, built on either a literature review or pre-existing data and a panel of experts undertaking a series of ‘rounds’ to identify, clarify, refine and finally to gain consensus. As the process is undertaken remotely, individuals can express their opinion without being influenced by others.

### Template generation

A template to kick off the first round and including 46 items was developed by a core group (SP, NN), based on pre-existing competencies catalogues for postgraduate training of geriatricians developed in Switzerland and in Belgium and the European Association Palliative Care (EAPC) white paper on palliative care education [11–13]. Duplicates of competences between the two were merged and rephrased by the core team.

The first questionnaire contained a list of 45 competencies that were divided in 11 chapters: principles of palliative care, symptom management, prognosis, decision-making, assisted dying, dying phase, grief, spirituality, communication, interdisciplinary team working, relatives and volunteers. Three demographic questions were also added for the participants: country, education in palliative care (number of days) and the clinical setting (acute or long-term care).

### Expert panel

At the start of the Delphi process, each panel member belonged to the SIG Palliative Care and/or to the SIG Education and Training of EUGMS.

### Delphi process

Members of both SIGs were contacted by e-mail. Members willing to contribute were sent one reminder for each Delphi round. Those who agreed to participate were provided with a link to an online questionnaire. Participants were asked to rate each item suggested in the template on a 5-point Likert Scale (from strongly disagree to strongly agree). Prior to begin of the Delphi process members of the core team members agreed on a threshold level (strongly agree and agree) of 80% to keep an item as definite in the list. Ratings between 50–80% of positive replies were discussed in between Delphi rounds among the Delphi core group and resent to raters in the next Delphi round, mostly in a version adapted according to comments. Items were deleted if less than 50% answered strongly agreed.

Members of interest groups were also invited to provide additional free-text responses if they had suggestions or amendments to any curriculum item or if there was additional content they wanted to propose. In response to participants’ suggestions, curriculum items were modified in a variety of ways that included: adjusting wording of items to improve clarity, readability and achievability of learning outcomes; this process of redrafting and refining curriculum items was undertaken by the authors through a series of online meetings.

The first round was running from January 2018 to February 2018 with a reminder sent end of February until end of March. The core group decided to repeat the same process during the next rounds until full consensus was reached among raters for all items with no limits of the number of Delphi rounds. The whole process of the modified Delphi was running until April 2019. Emails were sent individually and the use of an online questionnaire system ensured anonymity was preserved.

## Results

In total, 42 experts and members of the SIGs Palliative care and Education and Training, were contacted by mail and asked for their willingness to contribute to consensus building. All 42 experts agreed to participate in the Delphi-consensus. Twenty-three EuGMS experts were involved in the SIG Palliative care and 19 members represented the SIG Education and Training. All of them, except one, were trained geriatricians and were actively involved in medical care of older patients or/and in teaching.

### First round

Twenty-four of the 42 invited experts completed the first round of the online survey. They represent 12 European countries (Austria, Belgium, Denmark, Italy, France, Great-Britain, Ireland, Monaco, Norway, Poland, Spain, Switzerland) and the majority of them worked in acute geriatric units (*n* = 16). Their educational background in palliative care competences varied between 3 and 300 days of palliative care education during the Delphi process (see section Template generation).

Thirty-six competencies required modification due to written feedback from raters and were discussed by the core group. Following this revaluation the wording of 13 competencies and the content of 10 competencies were modified. Furthermore, two competencies and two whole chapters (assisted dying and decision-making) were merged according to feedback from raters. Some concepts such as the concept of dignity or spirituality were rated to not be specific enough. There was some overlap between geriatric and palliative care for symptom-management, such as confusion or constipation. Some symptoms were judged “too rare” (hiccups or ileus) to be included in a permanent curriculum. Geriatricians felt that they already had sufficient expertise in integrating volunteers and so we removed these competencies form the catalogue. After consideration of the ratings and feedback in the first Delphi round, the second survey version included 36 competencies.

### Second and third rounds

Twenty expert members of the 24 raters involved in round one completed the second survey round. At the end of this second round, one question was deleted: “Understanding the responses to loss and the influence of the response on decision-making of older people and/or their next of kin”. For three competencies the wording was modified. These modifications had the agreement of the participants during the last round (19 participants). The final version of core palliative care competences recommended by EuGMS for further translation into postgraduate training curricula for geriatric medicine included 35 competencies (see Table 1).

**Table 1:** Final version of the palliative care core compentencies for geriatricians

	Level of agreement (%)
Palliative care principles	
1. Understanding and applying the principles of palliative care. This means support people with life-limiting conditions to live as actively as possible until death with optimal quality of life and support families cope during illness	91.6
2. Diagnosing life-limiting condition, including prognostic factors, common symptoms and problems in the context of complex and multimorbid older people in geriatric medicine	91.6
3. Knowing when and how to consult specialists palliative care	82.6
4. Understanding illness trajectories, assessment of care needs and setting care goals	95.6
5. Managing the level of uncertainty related to assessment of patients with organ failure	82.6
6. Treating a person with life-limiting illness taking into account his own personality and life story.	94.7
Symptom assessment	
7. Knowing the principles of aetiology/ pathophysiology of pain in life-limiting illness and acting accordingly	86.3
8. Knowing the principles of aetiology/ pathophysiology of respiratory failure in life-limiting illness and acting accordingly	91.5
9. Knowing the principles of aetiology/ pathophysiology of constipation in life-limiting illness and acting accordingly	82.6
10. Knowing the principles of the pathophysiology of ileus in life-limiting illness and acting accordingly	81.2
11. Knowing the principles of the pathophysiology of nausea/ vomiting in life-limiting illness and acting accordingly	91.5
12. Knowing the principles of aetiology/ pathophysiology of cachexia/ anorexia in life-limiting illness and acting accordingly	80.9
13. Knowing the principles of aetiology/pathophysiology of delirium in life-limiting illness and acting accordingly	90.5
14. Knowing the principles of aetiology/ pathophysiology of fatigue/ asthenia in life-limiting illness and acting accordingly	81.0
15. Knowing the principles of the aetiology/ pathophysiology of depression in life-limiting illness and acting accordingly	81.2
16. Knowing the principles of aetiology/ pathophysiology of anxiety in life-limiting illness and acting accordingly	90.5
17. Able to properly administer drugs subcutaneously and/ off label in life-limiting illness	90.9
18. Understanding the concept, and ability to explain the procedure of palliative sedation	87.3
Prognosis issues	
19. Being aware of the uncertainty related to the medical prognosis	81.8
20. Knowing how to transmit the information that the patient needs confronted with a life-limiting disease	86.2
Decision-making at end-of-life	
21. Discussing and assessing patient’s concerns, wishes and expectations regarding care and decisions at the end of life with due regard for action when the person is unable to communicate or lacks mental capacity	90.9
22. Knowing the ethical and (country specific) legal principles of end-of-life decisions, including non-treatment decisions and medical assistance in dying (MAID)	94.7
23. Acting according to the ethical and legal principles regarding withdrawing and withholding of treatment	95.2
24. Acting according to the ethical and legal principles regarding use of artificial hydration and feeding	90.5
25. Acting according to the ethical and legal principles regarding Do Not Attempt Resuscitation Orders	90.4
Dying phase	
26. Recognizing the dying phase and identify the needs of the older person and act accordingly	94.8
27. Identifying and explaining the signs and symptoms of dying to the patient himself and to relatives	90.5
Grief	
28. Understanding that grief is a normal and appropriate response to loss	90.5
29. Recognizing symptoms of complicated grief and to distinguish depression from grief	90.5
Spirituality	
30. Understanding the nature of spirituality and recognise that everyone has a spiritual dimension and that for many people this may have a religious component	90.0
Communication	
31. Communicating and informing patients in a sensitive, timely and clear way about palliative care	95.0
32. Taking into account that communication of information which may influence decision-making and care planning at the end of life is an ongoing process	94.7
33. Assessing a patient’s response to the information shared, to check their understanding and respond appropriately	95.0
Interdisciplinary network	
34. Knowing the structure of the regional Palliative Care Network and having a list of contact details of the services available	80.0
Relatives	
35. Assessing the needs, strength and resilience of the family and social support system	85.7

## Discussion

The management of the last period of life in older patients is characterized by complex treatment decisions, challenging symptom management, manifold psychosocial problems and easily overlooked spiritual distress. Palliative care (PC) for older patients, however, is an essential building block for quality of living as well as for quality of dying, and the concepts and services related to its provision are part of the integrative care approach to health care that includes health promotion even in the presence of disease. The WHO definition of palliative care includes the provision of quality of life for patients and their families through the prevention/relief of suffering by means of early identification, comprehensive assessment and treatment of pain and other physical, psychosocial and spiritual suffering. Achieving these goals requires an integrative approach throughout societal structures, including appropriate policies, adequate access to treatment and interventions such as drug availability, education of both health care workers and public and implementation of generalist PC services at all levels of society. While not denying the reality of death, PC thus offers a positive approach for living life to the full even for older patients [14]. Early identification of patients in need of PC becomes crucial in particular for older patients with chronic conditions [15, 16].The Delphi process described in this publication enabled the development of a European specific core competency catalogue to improve competencies of geriatricians to enable them to guide their patients through the last period of life. The current version of the palliative core competency catalogue represents an important step in the development of effective palliative care education within the training of geriatricians, which is essential given the condition’s increasing relevance to twenty-first century healthcare. This catalogue equips geriatricians with skills mandatory to deliver person centered care to older patients until the end of their life.

A strength of this work is the fact that we reached consensus with a high level of agreement within a group of experts coming from a multitude of different countries and backgrounds. This enhances the likelihood that these competencies are relevant and applicable to the curriculum of geriatricians across Europe. However, it leaves space for nations to develop national curricula adapted to their specific needs and to the settings.

First limitation of the work is that experts may have scored competencies according to their own individual interests; however, experts were largely selected from groups with a special interest in this topic. Second limitation is that researcher influence on the formulation of the initial statements and it is possible that important competencies were missed in particular giving them a broad perspective on the needs of older patients living with frailty.

The challenges to implement the competencies across Europe are numerous: A lot of the competencies such as communication, decision-making should not be learned from lecturer but by methods that provide a potent stimulus for reflective practice such as simulated clinical interactions. Furthermore, given the multidisciplinary of geriatrics and palliative care, competencies for the other team members as nurses, psychologists, physiotherapists, occupational therapists should be developed in the near future. Also skills for caregivers and patients to deal with aspects of end of life should be developed in a future version.

Over the past few years, a number of groups have focused on integrating the principals of geriatrics and palliative care into primary care [17–21]. Geriatric medicine and palliative care are distinct but overlapping medical specialties [22]. They are both highly multiprofessional and interdisciplinary fields with patient- and family-centered activities aimed at improving quality of life, personal capabilities and social participation [23]. The synergies that result from joining these related specialties may serve as a role model for inter-specialty collaboration in health care. We also need a closer collaboration of the professional specialties geriatric medicine and palliative care, e.g., by organizing inter-specialty continuous professional Education. Considering the growing need of palliative care for older people, improving knowledge about palliative care principles and acquainting general palliative care skills of geriatricians and other health care professionals dealing with frail older patients is crucial. All health professionals need to incorporate holistic palliative care into their practice and need to know the right tools to identify the patient. Palliative medicine and geriatrics need to work together on behalf of our patients and families and not on behalf of our practice settings, our disciplinary tribes, or our specialties [24, 25]. To accomplish these goals, we will need to align strategies and then organize ourselves to work closely together to achieve them.

To facilitate national uptake following approval of the document by the EuGMS boards, translations of the recommended competencies must be developed in collaboration with the National partner societies of EuGMS and the UEMS-GMS. Each country should address the existing gaps in their existing training requirements.

## Appendix 1

### Palliative care principles

1. Understanding and applying the principles of palliative care. This means support people with life-limiting conditions to live as actively as possible until death with optimal quality of life and support families cope during illness.
2. Diagnosing life-limiting condition, including prognostic factors, common symptoms and problems in the context of complex and multimorbid older people in geriatric medicine.
3. Understanding the responses to loss and the influence of the response on decision-making of older people and/or their next of kin.
4. Knowing when and how to consult specialist palliative care.
5. Understanding illness trajectories, assessment of care needs and setting care goal.
6. Managing the level of uncertainty related to assessment of patients with organ failure.
7. Understanding the concept of dignity as it pertains to someone with a life-limiting illness and acting accordingly.

### Symptom assessment

8. Knowing the principles of aetiology/ pathophysiology of pain in life-limiting illness and acting accordingly.
9. Knowing the principles of aetiology/ pathophysiology of respiratory failure in life-limiting illness and acting accordingly.
10. Knowing the principles of aetiology/pathophysiology of constipation in life-limiting illness and acting accordingly.
11. Knowing the principles of the pathophysiology of ileus in life-limiting illness and acting accordingly.
12. Knowing the principles of the pathophysiology of nausea / vomiting in life-limiting illness and acting accordingly.
13. Knowing the principles of aetiology/pathophysiology of cachexia / anorexia in life-limiting illness and acting accordingly.
14. Knowing the principles of aetiology/pathophysiology of delirium in life-limiting illness and acting accordingly.
15. Knowing the principles of aetiology/ pathophysiology of fatigue / asthenia in life-limiting illness and acting accordingly.
16. Knowing the principles of aetiology/pathophysiology of hiccups.
17. Knowing the principles of the aetiology/pathophysiology of depression in life-limiting illness and acting accordingly.
18. Knowing the principles of aetiology/pathophysiology of anxiety in life-limiting illness and acting accordingly.
19. Knowing the essentials of pharmacology in palliative care of the symptoms listed.
20. Able to properly administer drugs subcutaneously and/of off label in life-limiting illness.
21. Understanding the concept, and ability to explain the procedure of palliative sedation.

### Prognosis issues

22. Being aware of the uncertainty related to the medical prognosis.
23. Knowing how to transmit the information that the patient needs confronted with a life-limiting disease.
24. Understanding the different decision-making processes (autonomous decision-making, informed consent, shared decision-making).

### Decision-making at end-of-life

25. Discussing and assessing patient’s concerns, wishes and expectations regarding care and decisions at the end of life with due regard for action when the person is unable to communicate or lacks mental capacity.
26. Explaining the concept of euthanasia and assisted suicide and acting according to the ethical and legal principles.
27. Acting according to the ethical and legal principles regarding withdrawing and withholding of treatment.
28. Acting according to the ethical and legal principles regarding use of artificial hydration and feeding.
29. Acting according to the ethical and legal principles regarding Do Not Attempt Resuscitation Orders.

### Assisted dying

30. Acting according to the ethical and legal principles regarding requests for euthanasia or physician-assisted suicide.

### Dying phase

31. Recognizing the dying phase.
32. Identifying the needs of the older person and act accordingly.
33. Identifying and explaining the signs and symptoms of dying to the patient himself and to relatives.

### Grief

34. Understanding that grief is a normal and appropriate response to loss.
35. Recognizing symptoms of complicated grief and to distinguish depression from grief.

### Spirituality

36. Understanding the nature of spirituality and recognise that everyone has a spiritual dimension and that for many people this may have a religious component.
37. Creating a safe space where they can name and address their spiritual needs.

### Communication

38. Communicating and informing patients in a sensitive, timely and clear way about palliative care.
39. Understanding that communication of information which fundamentally changes the persons understanding of their situation and or influences their decision-making or planning is an ongoing process and not a single event.
40. Assessing a patient’s response to the information shared, to check their understanding and respond appropriately.

### Interdisciplinary network

41. Knowing the structure of the regional Palliative Care Network and having a list of contact details of the services available.

### Relatives

42. Identifying and meeting the needs of family careers.
43. Assessing the needs, strength and resilience of the family and social support system.

### Volunteers

44. Understanding of the importance and value of volunteers´ contribution to palliative care.
45. Knowing the possible interventions of volunteers in palliative care.

## References

[1] Anderson DR, Mangen DJ, Grossmeier JJ, Staufacker MJ, Heinz BJ (2010). Comparing alternative methods of targeting potential high-cost individuals for chronic condition management. J Occup Environ Med.

[2] Lynn J, Forlini JH (2001). “Serious and complex illness” in quality improvement and policy reform for end-of-life care. J Gen Intern Med.

[3] Evers MM, Meier DE, Morrison RS (2002). Assessing differences in care needs and service utilization in geriatric palliative care patients. J Pain Symptom Manag.

[4] Pautex S, Curiale V, Pfisterer M, Rexach L, Ribbe M, Van Den Noortgate N (2010). A common definition of geriatric palliative medicine. J Am Geriatr Soc.

[5] Piers R, Pautex S, Curiale V, Pfisterer M, Van Nes MC, Rexach L (2010). Palliative care for the geriatric patient in Europe. Survey describing the services, policies, legislation, and associations. Z Gerontol Geriatr.

[6] Wayce CM (2012). Report of the Geriatrics-Hospice and Palliative Medicine Work Group: American Geriatrics Society and American Academy of Hospice and Palliative Medicine leadership collaboration. J Am Geriatr Soc.

[7] Cao Q, Lee TJ, Hayes SM, Nye AM, Hamrick I, Patil S (2015). Are geriatric medicine fellows prepared for the important skills of hospice and palliative care?. Am J Hosp Palliat Care.

[8] Roller-Wirnsberger R, Masud T, Vassallo M, Zobl M, Reiter R, Van Den Noortgate N (2019). European postgraduate curriculum in geriatric medicine developed using an international modified Delphi technique. Age Ageing.

[9] Diamond IR, Grant RC, Feldman BM, Pencharz PB, Ling SC, Moore AM (2014). Defining consensus: a systematic review recommends methodologic criteria for reporting of Delphi studies. J Clin Epidemiol.

[10] Masud T, Blundell A, Gordon AL, Mulpeter K, Roller R, Singler K (2014). European undergraduate curriculum in geriatric medicine developed using an international modified Delphi technique. Age Ageing.

[11] Checkliste und Wegleitung Lernziele und Kompetenzen Palliative Care für die Facharztausbildungen (2020). https://www.palliative.ch/fileadmin/user_upload/palliative/fachwelt/C_Fachgesellschaft/Fachgruppe_Aerzte/Dokumente_Fachgruppe/Checkliste_Empfehlungen_Lernziele_und_Kompetenzen_PC_fuer_Facharztausbildungen_DT.pdf. Accessed 18 Dec 2020

[12] Koninklijke academie voor geneeskunde van belgië (2020) https://kagb.login.paddlecms.net/sites/default/files/atoms/files/Advies20161022.pdf. Accessed 18 Dec 2020

[13] Gamondi C, Larkin P, Payne S (2013). Core competencies in palliative care: An EAPC white paper on palliative care education - Part 1. Eur J Palliate Care.

[14] Tiberini RRH (2015). Rehabilitative palliative care: enabling people to live fully until they die. A challenge for the 21st century.

[15] Gómez-Batiste X, Martínez-Muñoz M, Blay C, Amblàs J, Vila L, Costa X, Villanueva A, Espaulella J, Espinosa J, Figuerola M, Constante C (2013). Identifying patients with chronic conditions in need of palliative care in the general population: development of the NECPAL tool and preliminary prevalence rates in Catalonia. BMJ Support Palliat Care.

[16] Meier DE (2014). Focusing together on the needs of the sickest 5%, who drive half of all healthcare spending. J Am Geriatr Soc.

[17] Conway PH, Clancy C (2009). Transformation of health care at the front line. JAMA.

[18] Windhaber T, Koula ML, Ntzani E, Velivasi A, Rizos E, Doumas MT (2018). Educational strategies to train health care professionals across the education continuum on the process of frailty prevention and frailty management: a systematic review. Aging Clin Exp Res.

[19] Murray SA, Firth A, Schneider N, Van den Eynden B, Gomez-Batiste X, Brogaard T (2015). Promoting palliative care in the community: production of the primary palliative care toolkit by the European Association of Palliative Care Taskforce in primary palliative care. Palliat Med.

[20] Lakin JR, Koritsanszky LA, Cunningham R, Maloney FL, Neal BJ, Paladino J (2017). A systematic intervention to improve serious illness communication in primary care. Health Aff (Millwood).

[21] Bernacki R, Hutchings M, Vick J, Smith G, Paladino J, Lipsitz S (2015). Development of the Serious Illness Care Program: a randomised controlled trial of a palliative care communication intervention. BMJ Open.

[22] Pacala JT (2014). Is palliative care the "new" geriatrics? Wrong question–we're better together. J Am Geriatr Soc.

[23] Albers G, Froggatt K, Van den Block L, Gambassi G, Vanden Berghe P, Pautex S (2016). A qualitative exploration of the collaborative working between palliative care and geriatric medicine: Barriers and facilitators from a European perspective. BMC Palliat Care.

[24] Murray SA, Kendall M, Mitchell G, Moine S, Amblàs-Novellas J, Boyd K (2017). Palliative care from diagnosis to death. BMJ.

[25] Stiel S, Krause O, Berndt CS, Ewertowski H, Müller-Mundt G, Schneider N (2020). Caring for frail older patients in the last phase of life : challenges for general practitioners in the integration of geriatric and palliative care. Z Gerontol Geriatr.

